# Nitrosation and Nitration of Fulvic Acid, Peat and Coal with Nitric Acid

**DOI:** 10.1371/journal.pone.0154981

**Published:** 2016-05-13

**Authors:** Kevin A. Thorn, Larry G. Cox

**Affiliations:** U.S. Geological Survey, Denver Federal Center, MS 408, Denver, Colorado, 80225-0046, United States of America; Old Dominion Univ., UNITED STATES

## Abstract

Nitrohumic acids, produced from base extraction of coals and peats oxidized with nitric acid, have received considerable attention as soil ammendments in agriculture. The nitration chemistry however is incompletely understood. Moreover, there is a need to understand the reaction of nitric acid with natural organic matter (NOM) in general, in the context of a variety of environmental and biogeochemical processes. Suwannee River NOM, Suwannee River fulvic acid, and Pahokee Peat fulvic acid were treated with ^15^N-labeled nitric acid at concentrations ranging from 15% to 22% and analyzed by liquid and solid state ^15^N NMR spectroscopy. Bulk Pahokee peat and Illinois #6 coal were also treated with nitric acid, at 29% and 40% respectively, and analyzed by solid state ^15^N NMR spectroscopy. In addition to nitro groups from nitration of aromatic carbon, the ^15^N NMR spectra of all five samples exhibited peaks attributable to nitrosation reactions. These include nitrosophenol peaks in the peat fulvic acid and Suwannee River samples, from nitrosation of phenolic rings, and N-nitroso groups in the peat samples, from nitrosation of secondary amides or amines, the latter consistent with the peat samples having the highest naturally abundant nitrogen contents. Peaks attributable to Beckmann and secondary reactions of the initially formed oximes were present in all spectra, including primary amide, secondary amide, lactam, and nitrile nitrogens. The degree of secondary reaction product formation resulting from nitrosation reactions appeared to correlate inversely with the ^13^C aromaticities of the samples. The nitrosation reactions are most plausibly effected by nitrous acid formed from the reduction of nitric acid by oxidizable substrates in the NOM and coal samples.

## Introduction

Nitrohumic acids have received considerable attention as potential soil amendments for nitrogen fertilization and numerous other applications, such as plant biostimulants and chelating agents for metals [1–4]. They are typically produced from basic extraction of coals that have been subjected to oxidation with nitric acid. Howard provided an early review on coal oxidation studies [5]. Nitrohumic acids have also been extracted from peats and composts oxidized with nitric acid [4].

In the course of our work to obtain a ^15^N nuclear magnetic resonance (NMR) spectrum that represented a control reaction for nitration of natural organic matter (NOM), we reported that treatment of Suwannee River NOM with nitric acid resulted in both nitrosation and nitration reactions [6]. The occurrence of nitrosation reactions during nitric acid treatment of Illinois # 6 coal had previously been inferred from polarographic reduction studies of nitrohumic acids extracted from the coal by Green and Manahan [7], who also noted the inability of Infrared (IR) spectroscopy to unequivocally differentiate between nitro and nitroso groups in the nitrohumic acids. Early on, Fuchs and Charmbury et al. considered part of the nitrogen incorporated into nitric acid oxidized coal to be in the form of isonitroso (oxime) groups [8,9]. Here we extend the ^15^N NMR analyses to four additional samples reacted with nitric acid, the Suwannee River fulvic acid (SRFA), Pahokee Peat fulvic acid (PFA), bulk Pahokee Peat and Illinois #6 coal, demonstrating carbon nitrosation in each case, as well as N-nitrosation in the Pahokee peat samples.

Nitrous acid can be formed from the reduction of nitric acid by oxidizable substrates such as phenols, anisoles, and aniline derivatives in dilute aqueous solutions of nitric acid [10–13]. The nitrous acid can go on to react as a nitrosating agent. In the organic chemistry literature, the nitration of phenols via an in situ nitrosation-oxidation pathway has been documented with dilute aqueous nitric acid solutions at concentrations as low as 3% [10–13]. Reaction of the phenol with nitrous acid yields a nitrosophenol, which is then oxidized to a nitrophenol upon reaction with the residual nitric acid. The last step regenerates nitrous acid. Nitrous acid-catalyzed nitration reactions can also be effected by nitrous acid present as an impurity in the nitric acid. Additionally, a mechanism involving radical cation intermediates has also been demonstrated in nitrous acid-catalyzed nitration reactions [14]. Humic substances, peat and coal are all known to contain hydroxy-substituted aromatic structures, including hydroquinone and catechol moieties, which have the potential to serve as substrate sites for nitric and nitrous acid.

Identification of the structural forms of nitrogen in nitrohumic acids is desirable to understand the mode of operation and evaluate the effectiveness of nitrohumic acids in their various applications. How nitric acid is incorporated into coal and NOM is the first step in this determination. Before application of NMR to chemically unaltered materials became routine, analysis of oxidative degradation products was an important tool for the structural analysis of humic substances, providing significant information on the substitution patterns and degree of condensation of aromatic rings [15,16]. Nitric acid was frequently employed as the oxidant [17,18]. More recently, there has been an increasing interest in the role of abiotic reactions of inorganic nitrogen compounds with natural organic matter in a variety of environmental and biogeochemical processes. These include, for example, the fate of inorganic nitrogen contaminating acid forest soils through atmospheric deposition [19], co-denitrification and other coupled biotic-abiotic reactions [20,21], the atmospheric nitration of carbonaceous aerosols and humic like substances (HULIS) [22,23], the photochemistry of nitrogen dioxide at soil surfaces [24], and the reaction of nitrate with NOM to form nitrogenous disinfection byproducts during UV treatment of water [25]. There is a need for the systematic investigation of the mechanisms underlying the reaction of inorganic nitrogen compounds (ammonia, hydroxylamine, nitrite, nitrous acid, peroxynitrite, nitrate, nitric acid, nitric oxide, nitrogen dioxide, etc.) with NOM. Knowledge of how the inorganic nitrogen compounds react with NOM under controlled laboratory reactions is a prerequisite to understanding their interactions with NOM in more complicated environmental matrices. This study is the first detailed ^15^N NMR examination of the reaction of nitric acid with NOM. The concentrations of nitric acid used here do not reflect environmental conditions, but provide a starting point for an evaluation of the nitration chemistry.

## Materials and Methods

### Materials

Suwannee River NOM (SRNOM; 1R101N), Suwannee River fulvic acid (SRFA; Std. I; 1S101F), Pahokee Peat fulvic acid (PFA; Ref. I; 1R103F) and Pahokee Peat (Bulk Source; 1BS103P) were purchased from the International Humic Substances Society (IHSS). Natural abundance ^15^N and ^13^C NMR spectra of the IHSS samples, including ^13^C aromaticities (f_a_’s), have been reported [26,27]. Illinois #6 coal (high volume bituminous) was purchased as the powder from the Argonne Premium Coal Samples collection at Argonne National Laboratory [28,29]. Natural abundance ^15^N and ^13^C NMR spectra of the coal, including the ^13^C aromaticity, have also been reported [30,31]. Elemental analyses of the unreacted IHSS samples were provided by the IHSS and for the reacted IHSS samples by Huffman Hazen Laboratories, Golden, Colorado. Elemental analyses of the Illinois #6 Coal were provided by Argonne National Laboratory. Analyses of the reacted coal, and nitrogen content of the unreacted coal, were determined by Huffman Hazen Laboratories. The ^13^C aromaticity of the bulk peat was determined in this laboratory from solid state DP/MAS (direct polarization/magic angle spinning) ^13^C NMR (S1 Fig).

Nitric acid, 98 atom percent ^15^N, was purchased from ISOTEC as 40 weight % (7.8M) and from Aldrich as 10 N (51.3%) solutions. (Trade names are for identification purposes only and do not represent endorsement by the U.S. Geological Survey.)

### Reaction of Samples with Nitric Acid

For the SRFA and PFA, 200 mg of sample dissolved in 2 mL deionized water was charged with 1 mL of 40% nitric acid in an 11-mL vial with a Teflon screw cap cover, and stirred for 2 days (final nitric acid concentration of 15.2%; 3.0 M). The sample was then evaporated under a stream of nitrogen gas, dialyzed against a 100 Da MWCO cellulose acetate membrane, and freeze dried. The SRNOM was treated similarly but with a higher final concentration of nitric acid (22.2%; 3.9M) to accommodate the higher ash content of the SRNOM (Table 1). All three samples took on a noticeable orange color from the nitric acid treatment. The samples were dissolved in DMSO-d_6_ for liquid-state ^15^N NMR analysis. Three hundred mg of the bulk peat was slurried with 1 mL water and 2 g of 10 N nitric acid for 3 days in a Teflon bottle (final nitric acid concentration of 29%; 5.8M) and then air dried. The sample was dialyzed against a 500 Da MWCO cellulose acetate membrane and freeze dried. For the Illinois #6 coal, under a fume hood, in a round bottom reaction tube, 4 mL of nitric acid (40%) was added dropwise to 0.5 g of powdered coal at a rate to maintain control of the reaction and allow evolution of nitrogen dioxide, in a scaled down modification of the procedure of Green and Manahan [7]. The reaction slurry was passed through a 1 μm glass fiber filter, and the filter cake washed successively with deionized water, 1N HCl, and deionized water. The nitrated coal was then air dried.

**Table 1:** Elemental analyses for NOM samples before and after nitric acid treatment^a^.

	SRNOM	SRNOM	SRFA	SRFA	PFA	PFA	Peat	Peat	Coal	Coal
		HNO_3_		HNO_3_		HNO_3_		HNO_3_		HNO_3_
C	52.47	42.90	52.44	49.7	52.12	50.4	45.70	50.4	77.7	62.6
H	4.19	3.97	4.31	3.5	3.23	2.9	4.74	3.9	5.00	3.67
N	1.10	2.86	0.72	2.9	2.43	3.1	3.13	6.2	1.31	6.16
O	42.69	49.71	42.20	42.8	43.93	43.5	ND	43.7	13.5	ND
S	0.65	0.54	0.44	0.5	0.53	0.6	ND	0.8	4.83	ND
P	0.02	0.02	<0.01	ND	0.01	ND	ND	ND	ND	ND
Ash	7.0	4.4	0.46	0.7	1.58	1.8	15.0	15.0	15.5	7.53
N_R_/N_0_ ^b^		2.6		4.0		1.3		2.0		4.7
C/N	47.7	15.0	72.8	17.1	21.4	16.3	14.6	8.1	59.3	10.2

^a^ C, H, N, O, S and P reported on moisture free and ash free basis.

^b^ Ratio of elemental nitrogen content of reacted to unreacted sample.

### NMR Spectroscopy

Liquid-state ^15^N NMR spectra were recorded on a VARIAN Gemini 300 MHz NMR spectrometer at a resonant frequency of 30.4 MHz using a 10mm broadband probe. ACOUSTIC [32] ^15^N NMR spectra were recorded using a 35,111.7 Hz (1,154.3 ppm) spectral window, 0.2 sec acquisition time, 1.0 sec pulse delay, and tau delay of 0.1 msec. The inverse gated decoupled ^15^N NMR spectrum (gated decoupling without NOE (nuclear Overhauser enhancement)) of the SRNOM employed a 35,111.7 Hz (1,154.3 ppm) spectral window, 45° observe pulse, 0.5 sec acquisition time, and 2.0 sec pulse delay. DEPT ^15^N NMR spectra (distortionless enhancement by polarization transfer) were recorded with a 26,000 Hz spectral window, 0.2 sec acquisition time, 1.0 sec delay for proton relaxation, and ^1^J_NH_ of 90.0 Hz. Neat formamide (112.4 ppm) was used as an external reference standard. The ^15^N NMR chemical shifts are reported in ppm downfield of ammonia, taken as 0.0 ppm. The ACOUSTIC pulse sequence alleviates baseline roll from acoustic ringing. The baseline roll in the inverse gated decoupled spectra of the SRFA and PFA was pronounced, and for this reason only the inverse gated decoupled spectrum of the SRNOM is reported. With a pulse delay sufficient to eliminate differential saturation effects, and gated decoupling to eliminate differential NOE effects, the inverse gated decoupled spectrum provides a quantitative distribution of nitrogens.

Solid-state constant amplitude CP/MAS (cross polarization/magic angle spinning) ^15^N and ^13^C NMR spectra were recorded on a Chemagnetics CMX-200 spectrometer at resonant frequencies of 20.3 and 50.3 MHz, respectively, using a 7.5 mm low carbon background ceramic probe (zirconium pencil rotors). Acquisition parameters for ^15^N included a 30.0 kHz spectral window, 17.051 msec acquisition time, 1 msec contact time, 0.2 sec pulse delay, and spinning rate of 5.0 or 6.0 kHz. The ^15^N NMR chemical shifts are reported in ppm downfield of ammonia, taken as 0.0 ppm. The 1 msec contact time was chosen to achieve maximum signal intensity for the sp^3^ hybridized nitrogens.

Naturally abundant nitrogens do not contribute to the intensities of the peaks corresponding to the labeled nitrogens incorporated into the samples from nitric acid in the ^15^N NMR spectra that follow, because the signal from the label overwhelms the naturally abundant nitrogens. This can be ascertained from the relative number of transients required for both sets of signals to come up.

Acquisition parameters for the solid state DP/MAS ^13^C NMR spectrum of the untreated Pahokee bulk peat (S1 Fig) included a 30.0 kHz spectral window, 90° observe pulse, 17.051 msec acquisition time, 30 sec pulse delay, and spinning rate of 6.0 kHz. A comparison of the spectrum with one recorded at a pulse delay of 20 seconds showed no differences in intensities, indicating the pulse delay of 30 seconds is more than sufficient for quantitation. The FID was processed with 2 points of backwards linear prediction. Parameters for the CP/MAS ^13^C NMR spectra of the coal before and after nitric acid treatment (S2 Fig) included a 30.0 kHz spectral window, 17.051 msec acquisition time, 5 msec contact time, 0.5 sec pulse delay, and spinning rate of 6.0 kHz.

Aromaticities (^13^C f_a_) correspond to the area from 110 to 165 ppm divided by the total spectrum area as determined from quantitative liquid state ^13^C NMR spectra of the SRNOM, SRFA, and PFA [27] and the quantitative DP/MAS spectrum of the bulk peat (S1 Fig). In the latter case, areas under the first order spinning sidebands to the aromatic carbon peak were not significant enough to affect the measurement and so were not taken into account. The aromaticity reported for the coal corresponds to the area from 90 to 165 ppm divided by the total spectrum area [30].

## Results

### Elemental Analyses and ^13^C Aromaticities

Table 1 shows elemental analyses for the SRNOM, SRFA, PFA, and bulk peat before and after nitration. The largest increase in elemental nitrogen content occurred for the coal, from 1.31 to 6.16% (N_R_/N_0_ = 4.7; ratio of nitrogen content of reacted sample to unreacted sample) while the smallest increase occurred for the PFA, from 2.43 to 3.1% (N_R_/N_0_ = 1.3). Carbon to nitrogen (C/N) ratios decreased from 72.8 to 17.1 for the SRFA and from 21.4 to 16.3 for the PFA. The N_R_/N_0_ values compare to N_R_/N_0_ ratios of 1.5 to 4.5 and 2.2 to 5.8 for low rank South Brazilian coals treated with 11% and 25% nitric acid, respectively [1]. Because the SRFA, SRNOM, and PFA dissolve into the nitric acid solution, whereas the bulk peat and coal do not, by necessity reaction conditions differ for the samples. The increases in nitrogen content must be interpreted within this context.

Carbon-13 aromaticities of the samples are listed in Table 2 and indicate that the aromatic carbon contents of the samples increase in the order SRNOM & SRFA<PFA & bulk peat < coal. As the highest increases in nitrogen occur for the coal and SRFA, which are at the opposite ends of the carbon aromaticity range, no apparent relationship between aromaticity and increase in nitrogen content upon nitric acid treatment can be derived for this set of samples, at least under the treatment conditions employed. Other parameters that might correlate to relative increases in nitrogen but not analyzed here would include proton aromaticities, degree of aromatic ring condensation, and reduction potentials. Proton aromaticities, a measure of the degree of substitution on aromatic rings, would provide an estimate of the substrate sites on aromatic rings available for substitution by nitro or nitroso groups. The degree of aromatic ring condensation also is an indication of the sites on aromatic rings available for substitution by nitro or nitroso groups. The coal is expected to differ from the other samples in its concentration of condensed aromatic rings, having an average cluster size of 3 to 4 fused aromatic rings [30]. For Spanish lignite low rank coal, substitution of aromatic ring hydrogens by nitro groups was favorable when there were two adjacent aromatic hydrogens per ring [33]. Reduction potentials, a measure of the oxidizable substrates present, would affect the amount of nitrous acid formed and therefore the amount of nitrogen incorporated via nitrosation. Here, the main reason for determining increases in nitrogen was to establish the amount of label to be observed in the ^15^N NMR spectra.

**Table 2:** Carbon-13 NMR aromaticities (^13^C f_a_) of untreated samples^a^.

Sample	SRNOM ^b^	SRFA ^b^	PFA ^b^	Peat ^c^	Coal ^d^
^13^C f_a_	0.23	0.24	0.34	0.36	0.72

^a 13^C f_a_ = area from 110 to 165 ppm divided by total spectrum area.

^b^ From ref. [27].

^c^ S1 Fig.

^d^ From ref. [30]; area from 90 to 165ppm divided by total spectrum area.

The change in oxygen content for the nitrated coal was not determined but oxidation was confirmed by the increases of hydroxyl carbons at 80 ppm and carboxylic acid and other carbonyl carbons at 180 ppm in the solid state CP/MAS ^13^C NMR spectra (S2 Fig).

### Liquid State ^15^N NMR Spectra

**Fig 1:**
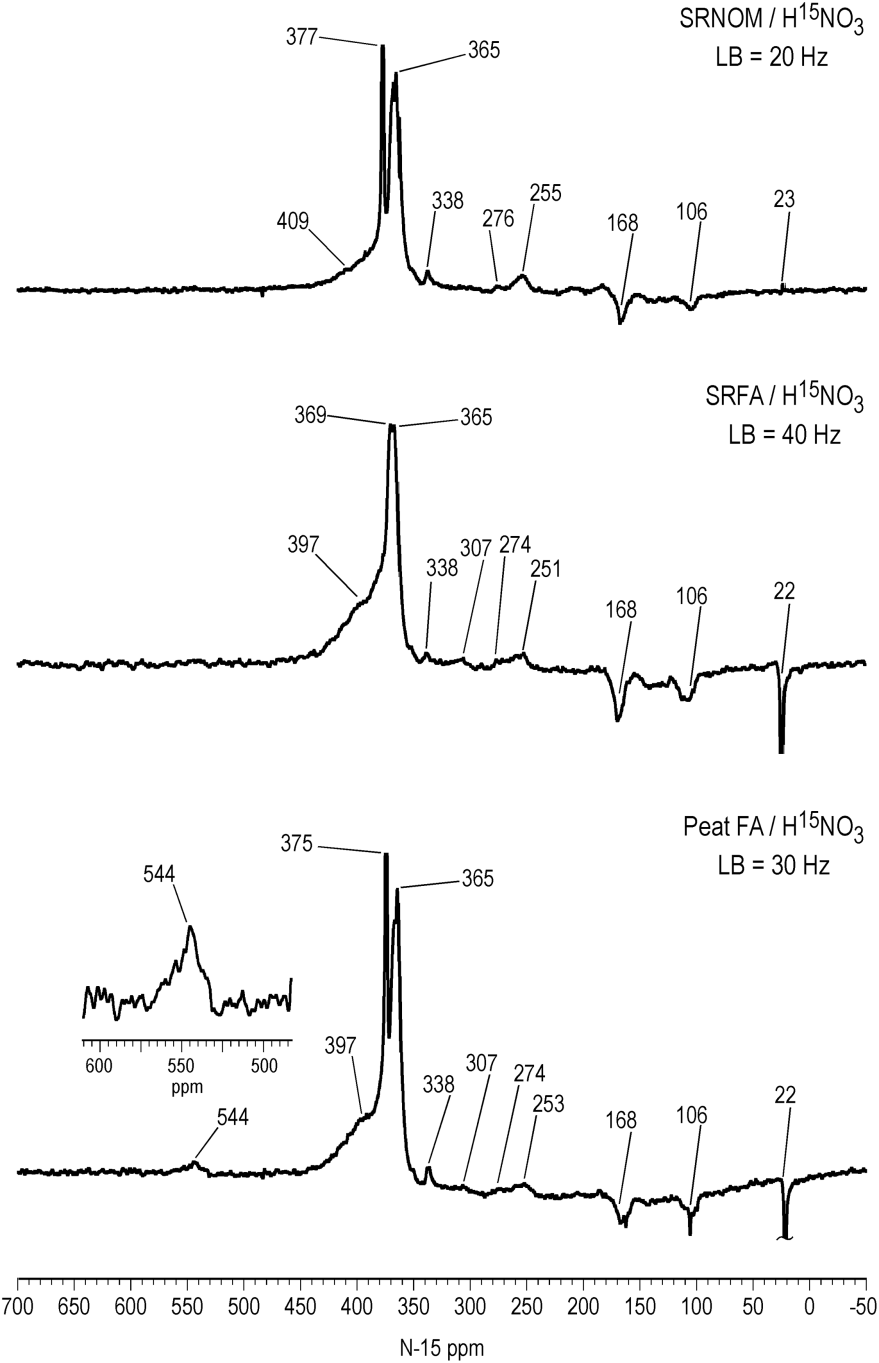
Liquid state ACOUSTIC N-15 NMR spectra of Suwannee River NOM, Suwannee River FA and Pahokee Peat FA treated with N-15 labeled nitric acid. LB = line broadening.

**Fig 2:**
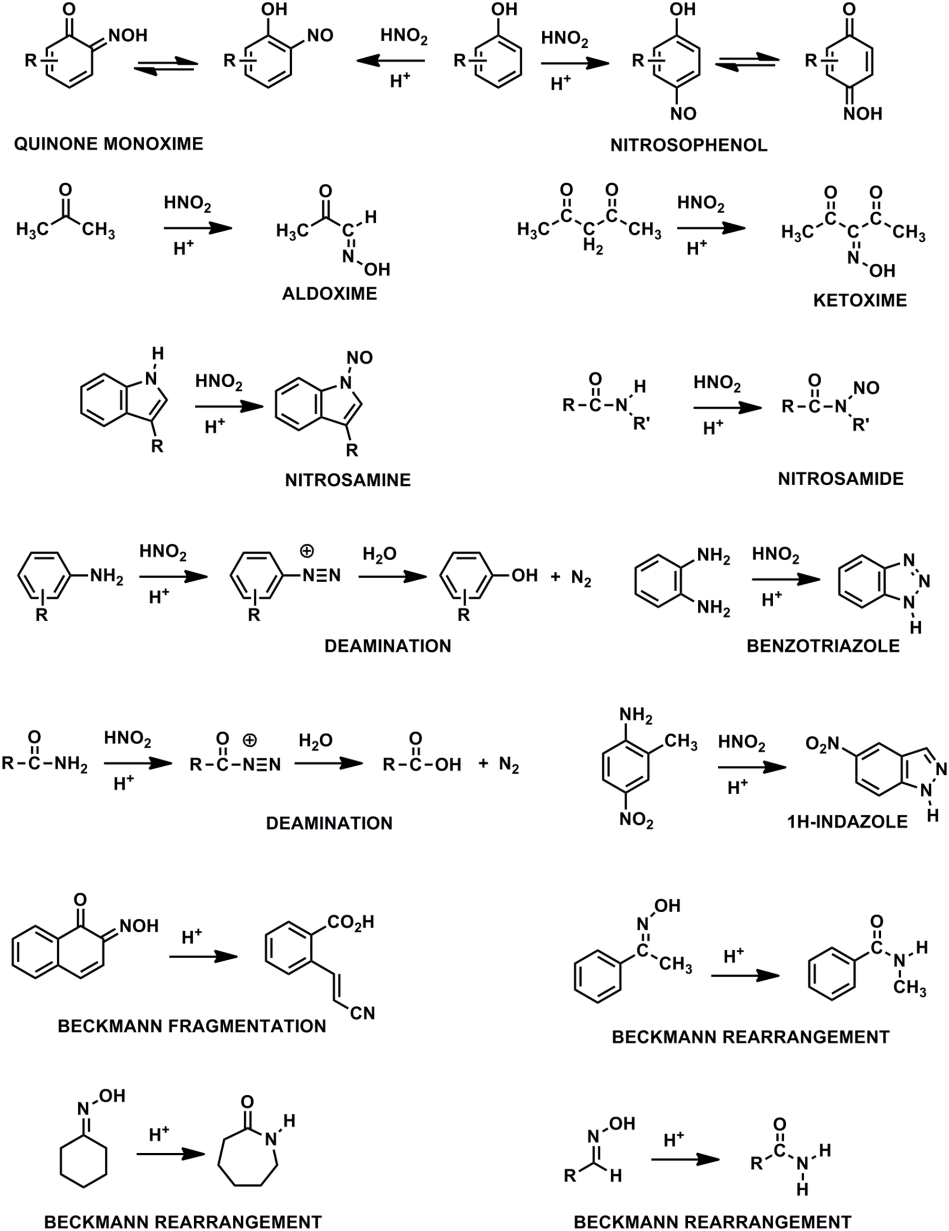
Nitrosation and Beckmann reactions. Modified from ref. [6].

**Fig 3:**
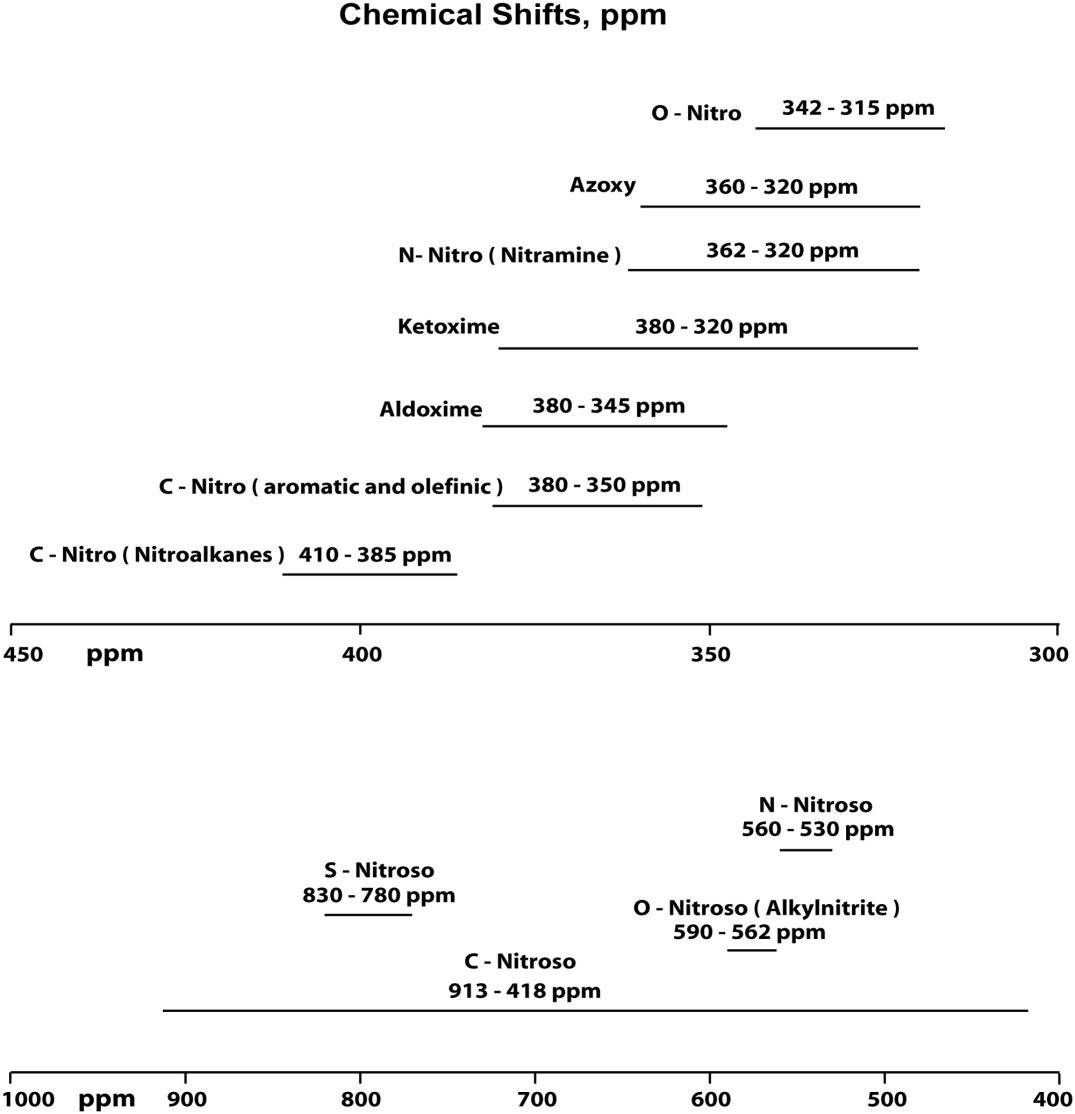
Nitrogen-15 NMR chemical shift ranges for oxime, nitro, and nitroso nitrogens on the ammonia scale. Reproduced from ref. [6].

Liquid-state ACOUSTIC ^15^N NMR spectra of the SRNOM, SRFA and PFA treated with nitric acid are shown in Fig 1. Retention of the NOE during acquisition of the spectra accounts for the negatively inverted peaks. The successful nitration of aromatic rings in the samples is evidenced by the aromatic nitro peaks at 365 ppm, the major peaks in the spectra. However, there is likely a contribution of ketoxime and aldoxime nitrogens to the peaks at 365 ppm, considering that nitrosation reactions occur concomitantly with the nitration reactions. Ketoximes and aldoximes result from nitrosation of activated methylene and methyl carbons, respectively (Fig 2). The ^15^N NMR chemical shifts of nitro, ketoxime and aldoxime nitrogens overlap (Fig 3 and S3 Fig). Discrete peaks corresponding to residual nitric acid not removed from the samples during dialysis are present at 377 ppm in the SRNOM and 375 ppm in the PFA. The remaining peaks in the spectra can be assigned to the reaction products of nitrosation reactions, including oximes, and to secondary reaction products from the initially formed oximes (Figs 2 and 3 and S3 Fig). We have observed a similar pattern of peaks in ^15^N NMR spectra of NOM samples reacted with hydroxylamine, directly nitrosated with sodium nitrite under acidic conditions, and reacted with sodium nitrate in the presence of unfiltered UV irradiation [6, 34, 35]. Assignments for the spectra are listed in Table 3. Some peaks have not been definitively assigned.

**Table 3:** Assignments for N-15 NMR spectra of NOM samples treated with nitric acid^a^.

Chemical Shift Range (ppm)	Peak Maxima (ppm)	Assignment	Assignment	Assignment
565–530	544 (PFA)	**N-nitrosamide**	**N-nitrosamine**	
	545 (Bulk Peat)			
430–390	397 (SRFA, PFA)	**Nitrosophenol (Quinone Monoxime)**	Aliphatic Nitro	
390–345	365 (SRNOM,SRFA, PFA)	**Nitro**	Ketoxime	Aldoxime
	369 (Bulk Peat)			
	367 (Coal)			
341–332	338 (SRNOM, SRFA,PFA)	Azoxy	O-Nitro	N-Nitro
	336 (Bulk Peat)	Ketoxime	Diazonium	
	333 (Coal)			
315–300	309 (Coal)	Pyridine	Indazole	Imine
	307 (SRFA, PFA)			
288–242	255 (SRNOM)	**Nitrile**		
	251 (SRFA)			
	254 (PFA)			
	250 (Bulk Peat)			
	253 (Coal)			
230–195	211 (SRNOM, SRFA)	**Nonprotonated Nitrogens**	Imidazole	Pyrazole
	210 (PFA)	Amidine	Imidate	
	209 (Bulk Peat, Coal)			
195–180	183 (SRNOM)	**Nonprotonated Nitrogens**	Isocyanide	
	184 (Coal)			
180–150	166 (SRNOM, PFA, Bulk Peat)	Hydroxamic Acid		
	165 (SRFA)			
	163 (Coal)			
	160 (PFA)			
150–120	137 (Coal)	**2° Amide**	**Lactam**	
	136 (PFA)			
	132 (SRNOM)			
	131 (SRFA)			
	127 (Bulk Peat)			
120–90	106 (SRNOM, SRFA, PFA)	**1° Amide**		

^a^ Most probable assignments are denoted in bold face.

All three spectra exhibit a nitrosophenol shoulder, from about 390 to 430 ppm, on the downfield side of the aromatic nitro peaksthat results from nitrosation of phenolic rings (Fig 1). These are especially well resolved in the spectra of the SRFA and PFA, at 397 ppm. The chemical shift of the nitrosophenol peak represents the tautomeric equilibrium position with its corresponding quinone monoxime form (Fig 2).

An estimate for the *minimum* amount of nitrosation versus nitration can be derived from electronic integration of the spectra from 430 to 345 ppm, considering the peak area from 430 to 390 ppm as nitrosophenols but *assuming* that the peak area from 390 to 345 ppm corresponds solely to nitro groups. In the case of the SRFA, which does not show residual nitric acid, the peak areas indicate 28% nitrosation and 72% nitration.

The ^15^N chemical shift range of aliphatic nitro groups, which would arise from *nitration* of activated methylene groups [36], is approximately 385 to 410 ppm (Fig 3). Although the presence of these groups cannot be excluded, the assignment of the peaks from 390 to 430 ppm as nitrosophenols is supported by their resemblance to the nitrosophenol peaks observed in spectra of other nitrosated samples [6, 35] and by the remainder of evidence for the occurrence of nitrosation reactions.

The peak at 544 ppm in the PFA corresponds to N-nitroso nitrogens, and may include both N-nitrosamide and N-nitrosamine adducts, from nitrosation of secondary amides (possibly including peptide nitrogens) and secondary amines, respectively. The PFA has the highest naturally abundant nitrogen content of the three samples in Fig 1, and therefore the highest number of potential sites for N-nitrosation. In general, primary amide and amine nitrogen would undergo decomposition to nitrogen gas upon diazotization with nitrous acid (Fig 2); the occurrence of these reactions cannot be ruled out. However, there are examples of heterocyclic pyrazole and triazole structures that result from nitrosation of primary aromatic amines, such as 2,3-naphthotriazole from 2,3-diaminonaphthalene, and 5-nitro-1H-indazole from 2-methyl-4-nitroaniline (Fig 2). The previously reported natural abundance ^15^N NMR spectrum for the PFA indicated the presence of both amide and heterocyclic nitrogen [26], with the latter possibly including indole nitrogens, likely substrate sites for N-nitrosamine formation. N-nitroso peaks were also observed in ^15^N NMR spectra of the Pahokee bulk peat and humic acid directly nitrosated with sodium nitrite [35].

All three spectra contain a peak at 338 ppm that appears to be a signature for treatment of NOM with nitric acid. The identity of the peak has not been established, but the chemical shift is within the range of several functionalities, including azoxy, O-nitro, N-nitro and ketoxime nitrogens. Azoxy compounds presumably would arise from condensation of phenylhydroxylamine and nitroso groups. The peak at 338 ppm was also observed in SRNOM reacted with sodium nitrate in the presence of unfiltered UV irradiation [6].

Peaks corresponding to nitriles, from Beckmann fragmentations of ketoximes and quinone monoximes, are present at 255, 251, and 253 ppm in the SRNOM, SRFA and PFA, respectively (Fig 1). Primary amides, from 90 to 120 ppm, with peak maxima at 106 ppm, are present in all three spectra and result from Beckmann fragmentations of ketoximes or Beckmann rearrangements of aldoximes (Fig 2). The assignment of the peaks at 168 ppm is uncertain. In ^15^N NMR spectra of NOM samples reacted with hydroxylamine, the peaks can be attributed to hydroxamic acids, from nucleophilic substitution of esters by hydroxylamine. Peaks corresponding to ammonium not removed in the dialysis are present at 23 ppm in the SRNOM and 22 ppm in the SRFA and PFA. Considering the acidic conditions of the nitric acid treatment, hydrolysis of secondary reaction products such as nitriles and primary amides is likely the predominant pathway for the production of ammonium, rather than reduction from nitrous acid via hydroxylamine. However, if the latter pathway occurs under the conditions of the nitric acid treatment, then formation of hydroxylamine could account for the peaks at 168 ppm.

Two other sets of very low intensity peaks are present in the spectra, including those at 276 ppm in the SRNOM and 274 ppm in the SRFA and PFA, on the downfield shoulders of the nitrile peaks. These are possibly attributable to benzotriazole nitrogens (S3 Fig). (A ^15^N NMR spectrum of Pahokee Peat humic acid directly nitrosated with sodium nitrate also showed a peak at 276 ppm [35].) The SRFA and PFA show peaks at 307 ppm (Fig 1), which are in the in the range of pyridine, indazole, and imine nitrogens (S3 Fig), among other sp^2^ hybridized heterocyclic nitrogens. The spectrum of the coal discussed in the next section also exhibits a peak at 309 ppm.

**Fig 4:**
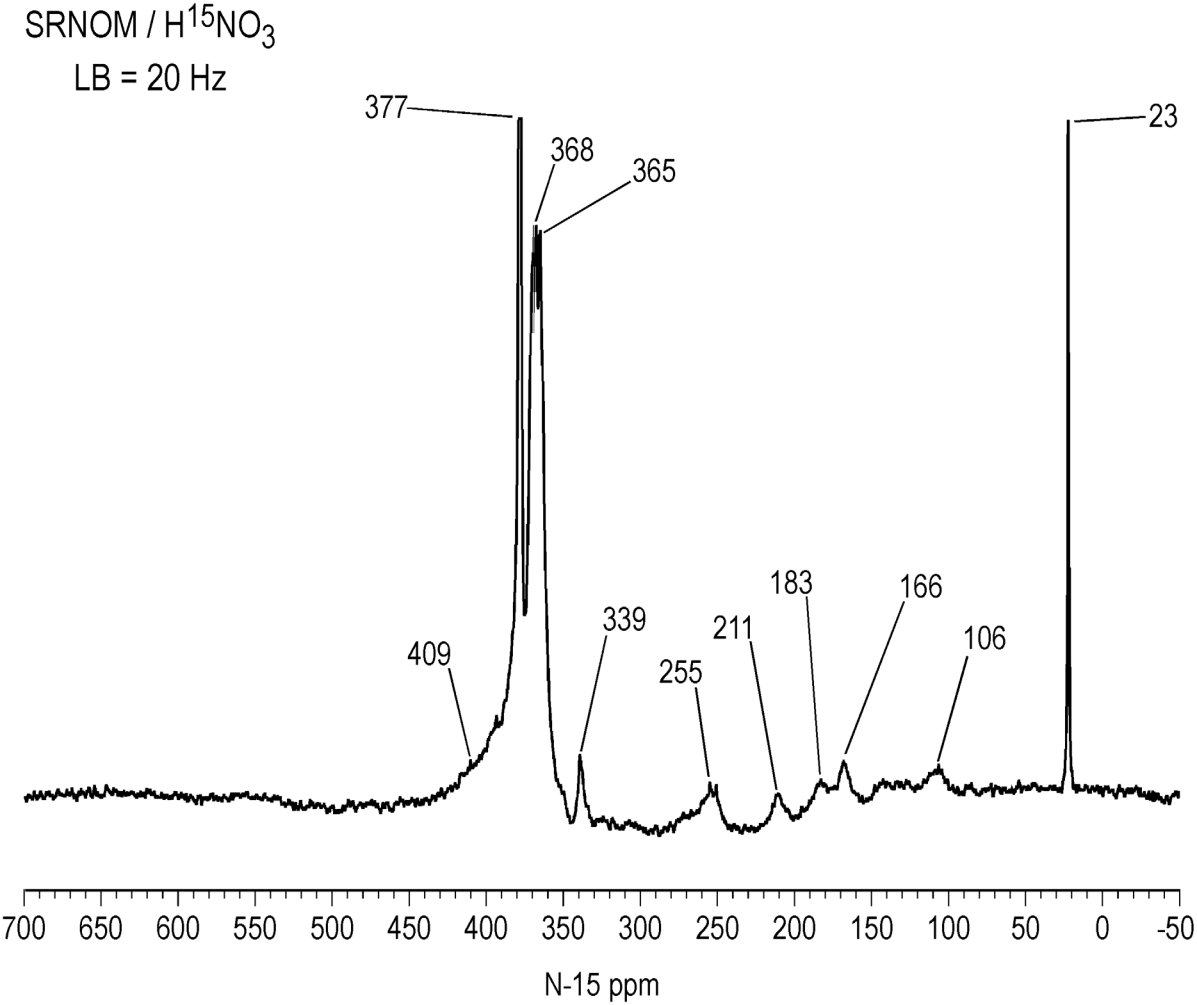
Liquid state inverse gated decoupled N-15 NMR spectrum of Suwannee River NOM treated with N-15 labeled nitric acid. LB = line broadening.

**Fig 5:**
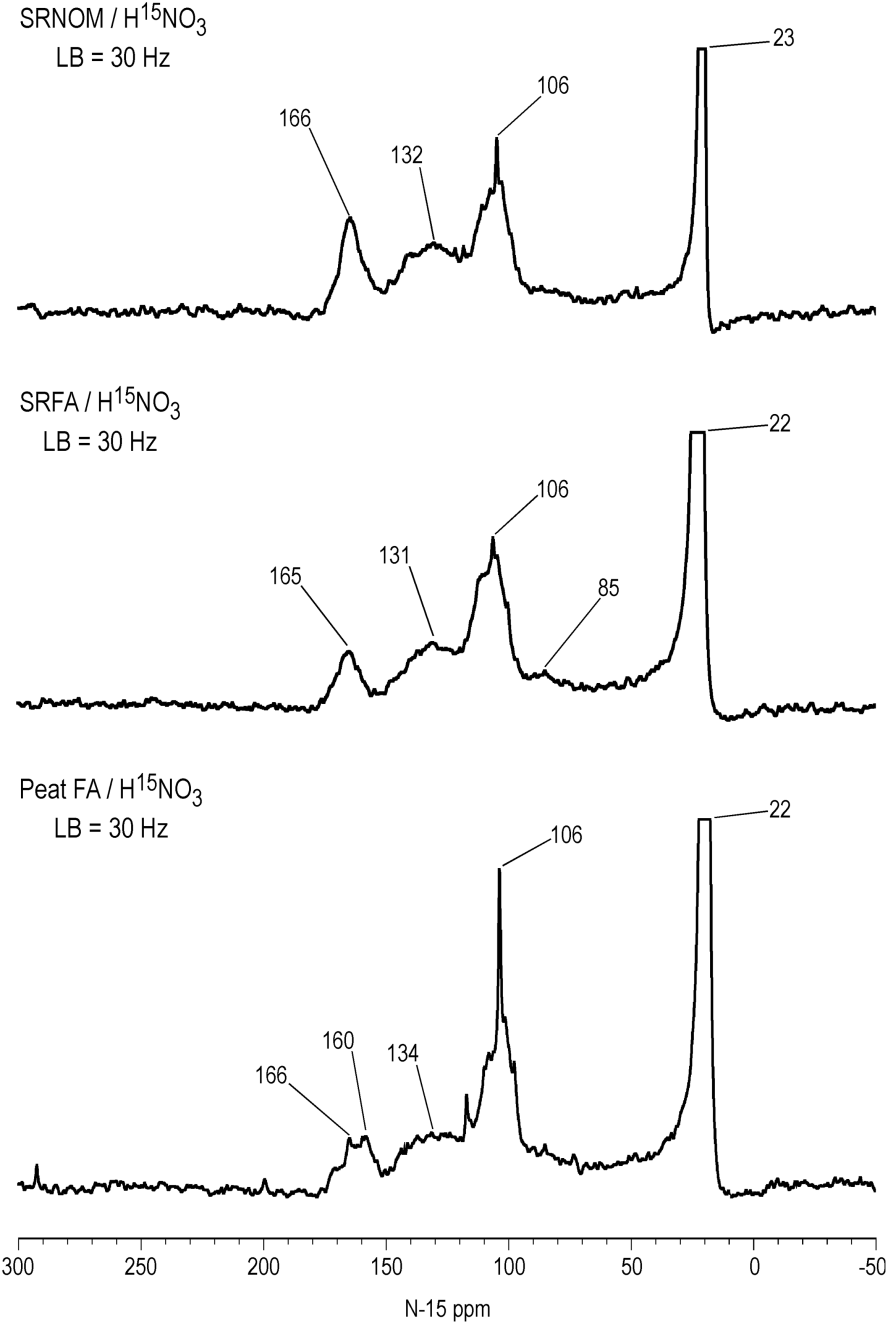
Liquid state DEPT N-15 NMR spectra of Suwannee River NOM, Suwannee River FA and Pahokee Peat FA treated with N-15 labeled nitric acid. LB = line broadening.

The inverse gated decoupled ^15^N NMR spectrum of the SRNOM (NOE eliminated) shows additional features in the region from 50 to 300 ppm, including distinct peaks at 183 ppm (assignment uncertain) and 211 ppm (possibly imidazole or pyrazole), and provides a quantitative distribution of nitrogens in the sample (Fig 4). There is essentially a continuum of peaks from 90 to 230 ppm that can be resolved into nitrogens bonded to protons from 90 to 180 ppm, and nitrogens not bonded to protons, from180 to 230 ppm, by comparison with the DEPT spectrum of Fig 5. The DEPT spectra show only nitrogens bonded to protons. They are similar for all three samples, showing three major peaks, excluding the residual ammonium peaks, from 90 to 120 ppm, 120 to 150 ppm, and 150 to 180 ppm (Fig 5). Further analysis not shown indicates that the nitrogens from 90 to 120 ppm are bonded to two protons and the nitrogens from 120 to 150 ppm and from 150 to 180 ppm are bonded to one proton. Thus the peaks centered at 106 ppm are confirmed as primary amides and the peaks centered at about 132 ppm as secondary amides and lactams. Again, the assignment for the peaks centered at about 166 ppm is uncertain. Through observation of these sp^3^ hybridized nitrogens corresponding to secondary reaction products, the recording of polarization transfer or indirect detection (e.g. ^1^H-^15^N gradient heteronuclear single quantum coherence (gHSQC)) spectra alone would suffice to confirm nitrosation.

### Solid State ^15^N NMR Spectra

**Fig 6:**
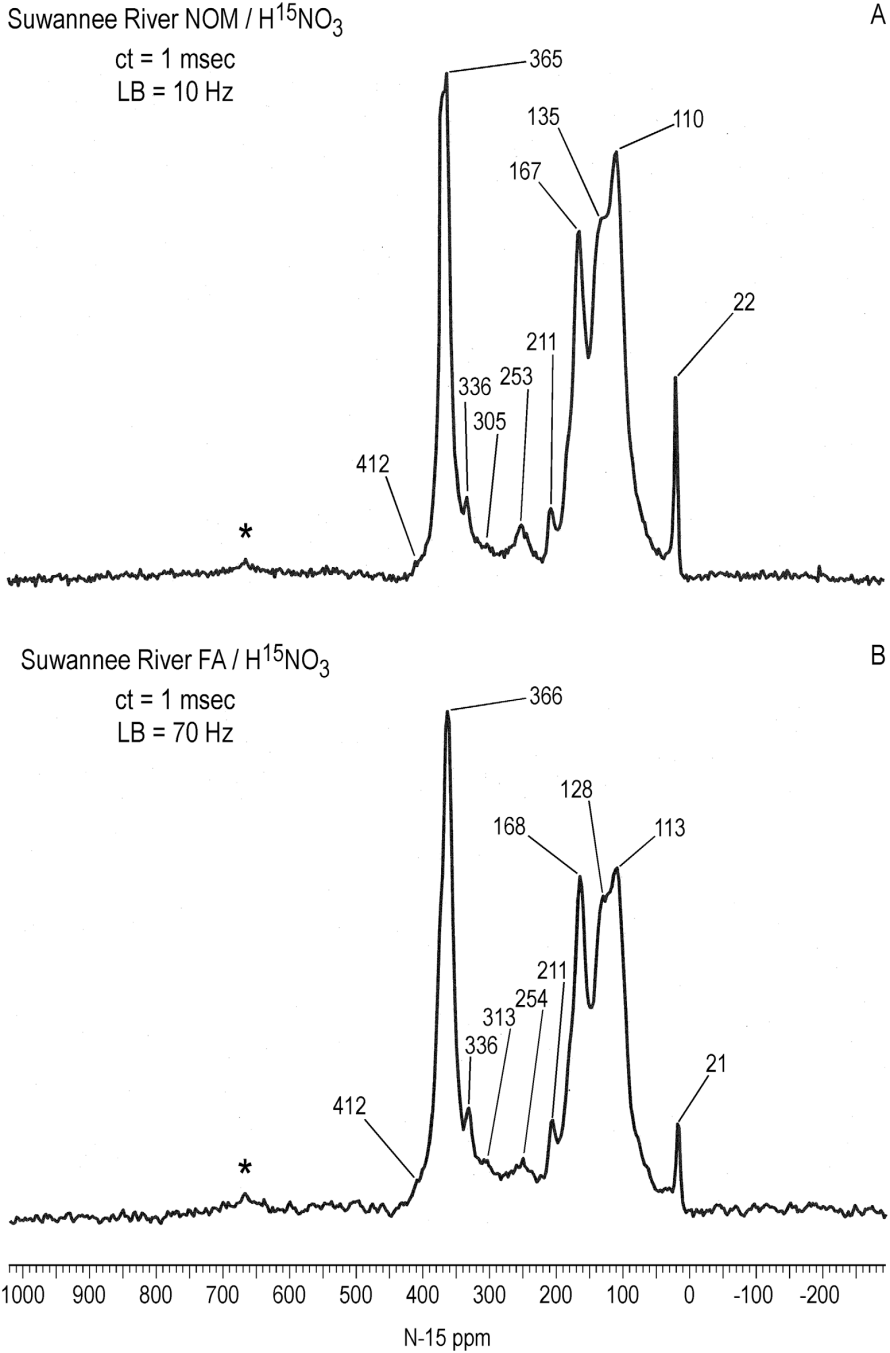
Solid state CP/MAS N-15 NMR spectra of Suwannee River NOM and SRFA treated with N-15 labeled nitric acid. ct = contact time. LB = line broadening. Spinning speed = 6 kHz. Asterisks denote spinning sidebands.

**Fig 7:**
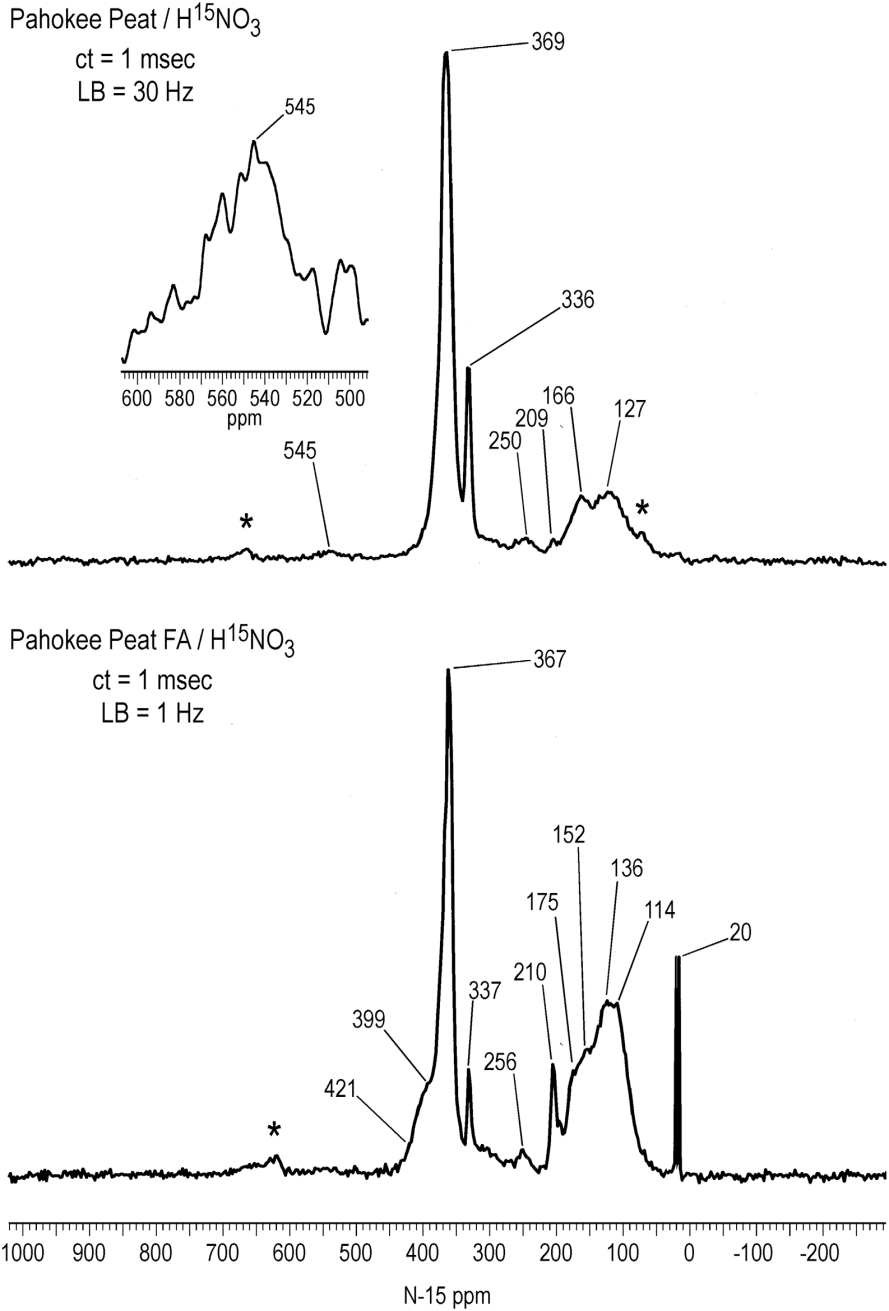
Solid state CP/MAS N-15 NMR spectra of bulk Pahokee Peat and Pahokee Peat fulvic acid treated with N-15 labeled nitric acid. ct = contact time. LB = line broadening. Spinning speeds for peat and peat fulvic acid were 6 kHz and 5 kHz, respectively. Asterisks denote spinning sidebands.

**Fig 8:**
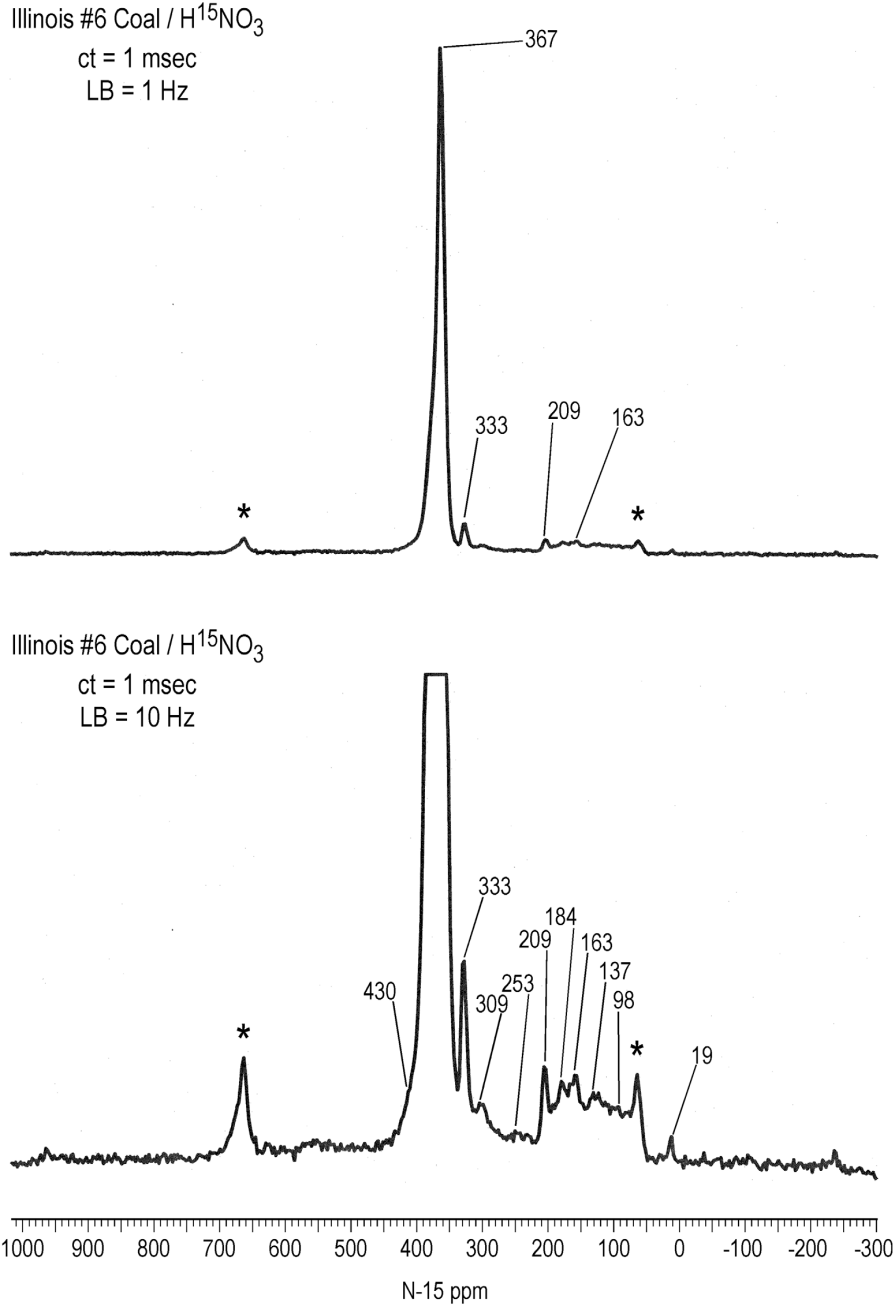
Solid state CP/MAS N-15 NMR spectra of Illinois #6 coal treated with N-15 labeled nitric acid. Bottom spectrum is vertical expansion. ct = contact time. LB = line broadening. Spinning speed = 6 kHz. Asterisks denote spinning sidebands.

The solid-state CP/MAS ^15^N NMR spectra of Figs 6–8 show all nitrogens incorporated into the samples from the labeled nitric acid, but do not represent a quantitative distribution of the nitrogens, as the spectra were acquired with a short contact time of 1 msec. Intensities of the nitrogens from 90 to 230 ppm are exaggerated in the CP/MAS experiment compared to the quantitative liquid-state inverse gated decoupled experiment, as evident from a comparison of the SRNOM spectra (Figs 4 and 6). Most advantageously, however, the solid state spectra allow a direct comparison of the soluble (SRNOM, SRFA, and PFA) and insoluble (peat, coal) nitric acid treated samples. The overall resemblance of the SRNOM and SRFA spectra reaffirm how similarly the two samples react with the nitric acid (Fig 6). The CP/MAS spectrum of the SRFA exhibits the distinct peak at 211 ppm also observed in the SRNOM spectra (Figs 4 and 6). The nitrated PFA and bulk peat are compared in Fig 7. The solid-state spectrum of the PFA shows some additional features not seen in the liquid-state spectra, such as the peak at 210 ppm. Conversely, the N-nitroso peak at 544 ppm in the PFA liquid-state spectrum of Fig 1 is not observed in the solid-state spectrum. The spectrum of the bulk peat does not exhibit a resolved nitrosophenol shoulder as does the PFA at 399 ppm, but the nitro/oxime/nitrosophenol peak does extend downfield to approximately 430 ppm. In the case of the bulk peat, however, the presence of the low intensity N-nitroso peak at 545 ppm and the peaks at 250, 209, 166 and 127 ppm corresponding to the secondary reaction products unequivocally indicate the occurrence of nitrosation reactions. The detection of the N-nitroso peak in the bulk peat is consistent with its high naturally abundant nitrogen content of 3.13%. The bulk peat also shows the peak at 336 ppm characteristic of nitric acid treatment. The PFA shows a greater proportion of secondary reaction products than the bulk peat (Fig 7).

Similar to the bulk peat, the spectrum of the nitrated Illinois #6 coal does not exhibit a well resolved nitrosophenol shoulder, but contains peaks at 253, 209,184, 163 and 137 ppm attributable to secondary reaction products from the nitrosation reactions (Fig 8). It also shows the characteristic peak at 333 ppm, an ammonium peak at 19 ppm, and a peak at 309 ppm (possibly pyridine, indazole or imine) that was also observed in the ACOUSTIC spectra of the SRFA and PFA (Fig 1). The coal has the lowest concentration of secondary reaction products, suggesting that nitro groups are the predominant form of incorporated nitrogen.

It is interesting to note that the proportion of nitrogens in the solid state spectra from 90 to 230 ppm that correspond to secondary products from nitrosation reactions decreases in the order SRNOM & SRFA > PFA & bulk peat > coal (Figs 6–8). This order follows the increase in ^13^C aromaticities of the samples (Table 2). In other words, the formation of secondary products from nitrosation reactions correlates inversely with the aromaticities of the samples. A possible explanation is that the greater the aliphatic carbon content, the greater the concentration of activated methylene and methyl carbons that are available for nitrosation and subsequent rearrangement. Again, other parameters that may be considered to rationalize the extent of secondary product formation would include proton aromaticities, degree of aromatic ring condensation, and reduction potentials of the samples.

## Discussion

In summary, the detection of N-nitrosamine, N-nitrosamide, nitrosophenol, nitrile, lactam, secondary amide, and primary amide nitrogens has provided evidence for the occurrence of nitrosation reactions. The most likely pathway to account for the nitrosation products observed in the ^15^N NMR spectra is reduction of nitric acid by the fulvic acids, peat and coal to nitrous acid, followed by nitrosation of the organic matter. Under the reaction conditions of these experiments, the nitrosophenols (quinone monoximes) observed in the spectra of the SRNOM and fulvic acids were stable enough to resist oxidation to nitro groups by the residual nitric acid. Furthermore, for a fraction of the oximes formed via nitrosation, rearrangement to secondary reaction products precluded oxidation to nitro groups by the nitric acid. The involvement of other reaction mechanisms cannot be ruled out, however. For example, the possibility of autoreduction of initially formed nitro groups to nitroso groups by organic matter was suggested by Green and Manahan in their investigation on nitration of coal [7]. Further studies will be required to resolve other aspects of both the nitration and nitrosation mechanisms, including the possibilities of direct nitration of activated methylene groups by nitric acid [36], the oxidation of ketoximes to nitro groups by residual nitric acid [36], and free radical reactions of nitric oxide and nitrogen dioxide with the NOM. Because the chemical shifts of nitro and oxime nitrogens overlap, accurately quantitating the degree of nitrosation versus nitration from the ^15^N NMR spectra of the nitric acid treated samples is not possible. As we noted previously, nitro and oxime groups are distinguishable by their ^17^O NMR chemical shifts, and thus ^17^O NMR analysis of NOM samples nitrated with ^17^O labeled nitric acid would be a possible approach to resolve this question [6], pending the availability of suitably enriched reagent. NMR spectral editing techniques may also help to resolve nitro groups from oximes, e.g. the SPIDER sequence [37]. Further studies will also be necessary to confirm the assignments of the ^15^N NMR peaks with maxima at 336–338, 307, 209–211, and 163–168 ppm that have been revealed to be diagnostic for treatment of NOM with nitric acid.

The aromatic nitro groups formed during the nitric acid treatment of coal and peat are susceptible to nucleophilic substitution by hydroxide ion during the subsequent base extraction of the nitrohumic acids from the coal, depending on the strength of the extractant employed [38]. There is potential for the released nitrite to undergo both oxidation and reduction, and for the reduced forms to condense with the carbonyl functionality of the organic matter, somewhat analogous to what occurs during the alkaline hydrolysis of 2,4,6-trinitrotoluene [39]. The question of what are the chemical alteration reactions that occur during the extraction process, and what are the structural forms of nitrogen in the final nitrohumic acid product, in particular when ammonium hydroxide is used as the extractant, can be addressed through further ^15^N NMR analyses.

Nitric acid, nitrate, ammonia, ammonium, nitrogen dioxide and organic nitrogen are among the forms of nitrogen taken into account when combined wet and dry nitrogen deposition budgets to soils are constructed [40]. The minimum concentrations at which nitric acid can directly react with organic matter, and undergo reduction to nitrous acid, in the litter layers of acid forest soils and surface soils of peatlands, have not been determined. Nitration and nitrosation of organic matter in this manner has not been evaluated as a possible mechanism contributing to the abiotic immobilization of nitrogen by soil organic matter. If nitro and nitroso groups can be incorporated into NOM under this scenario, they would be subject to microbial and abiotic reduction.

## Conclusions

Nitrogen-15 NMR has confirmed that treatment of the Suwannee River, Pahokee peat, and Illinois #6 coal samples with nitric acid results in both nitration and nitrosation reactions, including N-nitrosation of the peat samples that contain relatively high naturally abundant nitrogen contents. Further research will be required to confirm the mechanisms leading to the nitrosation reactions as well as the identities of the secondary reaction products from both nitration and nitrosation. The diversity of the structural forms of nitrogen incorporated into the samples from nitric acid is much greater than previously realized. Further studies will also be necessary to determine the structural forms of nitrogen in the base extracts of nitric acid treated coals and peat.

## Supporting Information

S1 Fig Solid state C-13 Direct Polarization/Magic Angle Spinning (DP/MAS) NMR spectrum of bulk Pahokee Peat. pd = pulse delay. ss = spinning speed. LB = line broadening.
(TIF)

S2 Fig Solid state C-13 Cross Polarization/Magic Angle Spinning (CP/MAS) NMR spectra of Illinois #6 Argonne Premium Coal before and after oxidation with nitric acid. ct = contact time. ss = spinning speed. LB = line broadening.
(TIF)

S3 Fig Nitrogen-15 NMR chemical shifts of model nitro, nitroso, oxime, and secondary reaction product compounds. Chemical shifts are in ppm on the ammonia scale. Separate resonances are observed for the Z and E isomers of ketoximes. In general, the E isomers of ketoximes are deshielded with respect to the Z isomers, whereas the reverse is true for aldoximes. Chemical shifts are from A (Levy and Lichter, 1979), B (Witanowski et al., 1993), C (Berger et al., 1997), D (Thorn et al., 1992), and E (determined in this laboratory). Solvents = a (acetone), b (benzene), c (chloroform), d (dimethyl sulfoxide), m (methylene chloride), s (solid state), and w (water). Berger S, Braun S, Kalinowski H-O, NMR spectroscopy of the non-metallic elements, John Wiley & Sons, 1997; Levy G, Lichter RL, Nitrogen-15 nuclear magnetic resonance spectroscopy, John Wiley & Sons, 1979; Thorn KA, Arterburn JB, Mikita MA, ^15^N and ^13^C NMR investigation of hydroxylamine-derivatized humic substances, Environ Sci Technol. 1992 (26), 107–116; Witanowski M, Stefaniak L, Webb G, Nitrogen NMR Spectroscopy, Academic Press, 1993.
(PDF)

## Abbreviations

CP/MAS: cross polarization/magic angle spinning
DEPT: distortionless enhancement by polarization transfer
DP/MAS: direct polarization/ magic angle spinning
IR: infrared
NMR: nuclear magnetic resonance
NOE: nuclear Overhauser enhancement
NOM: natural organic matter
