# Supplementary material for: Nitrosation and Nitration of Fulvic Acid, Peat and Coal with Nitric Acid
Source: PLoS One. 2016 May 13;11(5):e0154981. doi: 10.1371/journal.pone.0154981 (PMC4866739; doi:10.1371/journal.pone.0154981)
Supplement: S3 Fig — Chemical shifts are in ppm on the ammonia scale. Separate resonances are observed for the Z and E isomers of ketoximes. In general, the E isomers of ketoximes are deshielded with respect to the Z isomers, whereas the reverse is true for aldoximes. Chemical shifts are from A (Levy and Lichter, 1979), B (Witanowski et al., 1993), C (Berger et al., 1997), D (Thorn et al., 1992), and E (determined in this laboratory). Solvents = a (acetone), b (benzene), c (chloroform), d (dimethyl sulfoxide), m (methylene chloride), s (solid state), and w (water). Berger S, Braun S, Kalinowski H-O, NMR spectroscopy of the non-metallic elements, John Wiley & Sons, 1997; Levy G, Lichter RL, Nitrogen-15 nuclear magnetic resonance spectroscopy, John Wiley & Sons, 1979; Thorn KA, Arterburn JB, Mikita MA, 15N and 13C NMR investigation of hydroxylamine-derivatized humic substances, Environ Sci Technol. 1992 (26), 107–116; Witanowski M, Stefaniak L, Webb G, Nitrogen NMR Spectroscopy, Academic Press, 1993. (PDF) [file pone.0154981.s003.pdf]

N-Nitrosamide

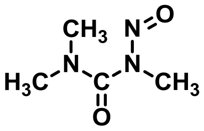

562 (B,c)

N-Nitrosamine

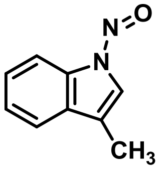

555 (A,b)

N-Nitrosamine

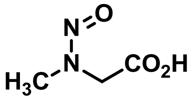

E=540 Z=534 (C,m)

Quinone Monoxime

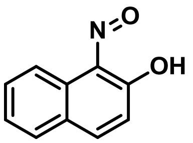

425 (D,d)

Quinone Monoxime

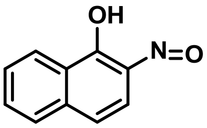

418 (D,d)

Benzofurazan

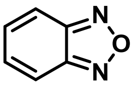

416 (C,d)

Quinone Dioxime

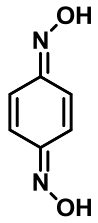

377 (D,d)

Nitroaromatic

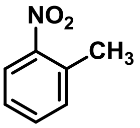

375 (A)

Nitroaromatic

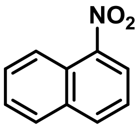

374 (C,c)

Benzotriazole

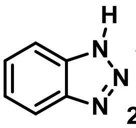

276
368
276 (C,d)

Nitramine

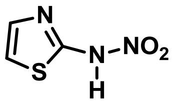

364 (B,d)

Ketoxime

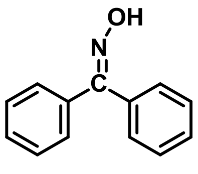

362 (D,d)

Azoxy

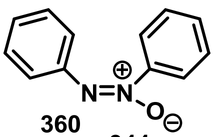

360
344
(A)

Nitramine

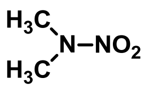

356 (C,a)

Ketoxime

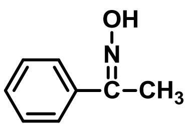

E=356 Z=325 (D,d)

Aldoxime

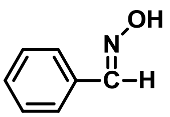

E=354 (A,d)

Aldoxime

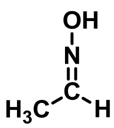

Z=350 E=346 (B,w)

Diazonium

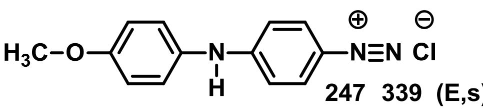

247 339 (E,s)

O-Nitro

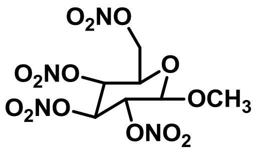

336-342 (C,a)

**Azoxy**

328

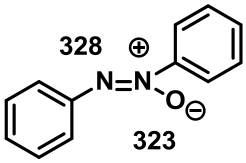


323 (B,d)

**Pyridine**

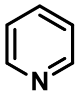


317 (B,d)

**Indazole**

180

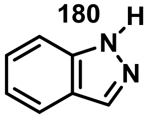


315 (C,a)

**Nitrile**

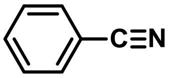


256 (D,d)

**Nitrile**

$H_3C-C\equiv N$

243 (D,d)

**Imidazole**

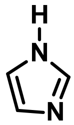


211 (B,d)

**Hydroxamic Acid**

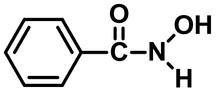


166 (D,d)

**Hydroxamic Acid**

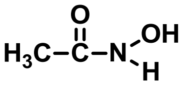


166 (D,d)

**2ndary Amide**

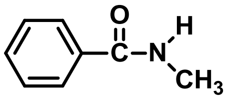


131 (D,d)

**Lactam**

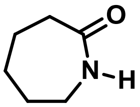


126 (B,w)

**Lactam**

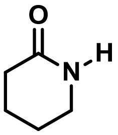


122 (B,w)

**Primary Amide**

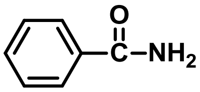


105 (D,d)
